# Report of the Diseases of Edinburgh for December, 1808

**Published:** 1809-02

**Authors:** John Roberton

**Affiliations:** Surgeon


### *Report of the Diseases of Edinburgh for December,* 1808. By JOHN ROBERTON, Surgeon.

This month commenced with wet unpleasant weather, which, with very little interruption, continued till about the middle of it. We then had a very heavy fall of snow, accompanied by unusually severe blasts of wind, which, from time to time, continued till near the end of the month, when the weather became soft, and, by the end of it, the snow was almost entirely dissolved. We scarcely experienced any frost during the month, except for one or two days previous to the fall of snow. Independently of this this state of the weather, the barometer in general stood high, till within a day or two of the end of the month, when it fell considerably. The general range of the thermometer was from about 35 to 40.

The prevailing complaints have been very numerous, and in greater variety than I ever recollect to have seen them in almost any month within my recollection. Those, however, which proved most destructive of life, were of an inflammatory nature, and fevers of the typhoid type, of a very bad kind. For the first of these, bleeding sometimes to a great extent, was of course found to be the best remedy, with the occasional exhibition of saline purgative medicines. It is a fortunate circumstance, considering the prevalence of inflammatory affections in this part of the world, that, in general, bleeding can be employed to a very great extent, without, as is often the case in London for instance, or indeed in any very large city, where the air is vitiated by the various processes carrying on in such places; independently of this too, there probably may be something immediately connected with the state of the climate between Edinburgh and London, which contributes to produce this difference of effect. The fevers I have alluded to, are treated in various ways, either according to the *whim*, or the degree of reflection and discernment of the medical attendant. A great number of medical men, particularly in this part of the world, have *lately* taken it into their heads, that this, as well as many other diseases, were to be cured by the repeated administration of purgative medicines alone. This practice is certainly of the greatest service in the removal of such diseases as principally exist in consequence of previous inattention to the state of the bowels, but, like every other mode of practice, when indiscriminately applied, (which I fear is too often the case in the practice I have just alluded to) it must be attended with very bad consequences. It must appear to every one, that the regulation of all natural evacuations, and the suppression of all morbid discharges, is absolutely necessary to the preservation of health; but the carrying of the one beyond what is proper, and the treating of the other with ineffectual timidity, are equally wrong. I am sorry to say, that in the treatment of diseases in general, the public are too often led to adopt plans recommended more from the generally imagined abilities of their proposer, than from any real merit which the plan itself possesses; while the most valuable practice is slowly, and with fear and trembling, adopted. Indeed, I cannot help regretting that the

 practitioner practitioner of physic as well as surgery, in every part of the world, is too often more indebted for his success to the *weakness of others than any merit of his own.* M. Morveau justly and beautifully remarks, that "it would be deserting the cause of humanity merely to deplore the lukewarmness with which the most useful truths are received, and the difficulty of overturning the established routine by which error is transmitted from age to age." This, I am sorry to say, for the reasons above assigned, there is too much reason to regret in the practical part of the medical profession, and it is the most sincere of my wishes to avoid the imputation.

I have before observed that the complaints of the present month have been very numerous; indeed, it would be unnecessary and certainly very uninteresting to detail them individually; I may, however, observe, that those I have above alluded to, seem to be the most destructive of life. Those of an inferior order seem principally to be cynanche tonsillaris, eruptions, or rather small abscess, on various parts of the body, rheumatism, catarrh, &c. For the removal of the first of these, blisters applied to the throat; gargles, into the composition of which some of the acids enter, with the exhibition of purgative medicines, seem extremely proper. The next, not being very alarming, either in their appearance or consequences, seldom require any thing more than gentle doses of physic, with the application of any kind of astringent wash, for their entire removal. Those cases of rheumatism and catarrh, which occur in practice, are in general removed by the most common remedies; the rheumatism by remedies of a gently sudorific nature, the catarrh by purgative medicines and confinement to the house for a few days.

*No. 4, St. James's Street.*

